# The transcriptome landscape of the carcinogenic treatment response in the blind mole rat: insights into cancer resistance mechanisms

**DOI:** 10.1186/s12864-018-5417-z

**Published:** 2019-01-08

**Authors:** Robert Altwasser, Arnon Paz, Abraham Korol, Irena Manov, Aaron Avivi, Imad Shams

**Affiliations:** 1 Institute of Evolution, University of Haifa, Haifa, Israel; 2 Department of Evolutionary and Environmental Biology, University of Haifa, Haifa, Israel

**Keywords:** Cancer, Fanconi anemia, Spalax, Cancer-resistance, Extracellular matrix

## Abstract

**Background:**

*Spalax*, the blind mole rat, developed an extraordinary cancer resistance during 40 million years of evolution in a subterranean, hypoxic, thus DNA damaging, habitat. In 50 years of *Spalax* research, no spontaneous cancer development has been observed. The mechanisms underlying this resistance are still not clarified. We investigated the genetic difference between *Spalax* and mice that might enable the *Spalax* relative resistance to cancer development. We compared *Spalax* and mice responses to a treatment with the carcinogen 3-Methylcholantrene, as a model to assess *Spalax*’ cancer-resistance.

**Results:**

We compared RNA-Seq data of untreated *Spalax* to *Spalax* with a tumor and identified a high number of differentially expressed genes. We filtered these genes by their expression in tolerant *Spalax* that resisted the 3MCA, and in mice, and found 25 genes with a consistent expression pattern in the samples susceptible to cancer among species.

Contrasting the expressed genes in *Spalax* with benign granulomas to those in *Spalax* with malignant fibrosarcomas elucidated significant differences in several pathways, mainly related to the extracellular matrix and the immune system. We found a central cluster of ECM genes that differ greatly between conditions.

Further analysis of these genes revealed potential microRNA targets.

We also found higher levels of gene expression of some DNA repair pathways in *Spalax* than in other murines, like the majority of Fanconi Anemia pathway.

**Conclusion:**

The comparison of the treated with the untreated tissue revealed a regulatory complex that might give an answer how *Spalax* is able to restrict the tumor growth. By remodeling the extracellular matrix, the possible growth is limited, and the proliferation of cancer cells was potentially prevented. We hypothesize that this regulatory cluster plays a major role in the cancer resistance of *Spalax*. Furthermore, we identified 25 additional candidate genes that showed a distinct expression pattern in untreated or tolerant *Spalax* compared to animals that developed a developed either a benign or malignant tumor. While further study is necessary, we believe that these genes may serve as candidate markers in cancer detection.

**Electronic supplementary material:**

The online version of this article (10.1186/s12864-018-5417-z) contains supplementary material, which is available to authorized users.

## Background

The long-lived (> 20 years) subterranean rodent *Spalax* (genus *Spalax ehrenbergi* complex) is a solitary inhabitant of sealed underground burrows in the Eastern Mediterranean region [1]. While these tunnels protect the animals from predation and climatic extremes, they are also prone to sharp drops in O_2_ levels, which can reach ~ 7% in the rainy season [2]. During these periods, *Spalax* performs intensive digging work under low O_2_ conditions and rapid re-oxygenation. *Spalax* displays an extraordinary hypoxia tolerance that has evolved over 40 million years of the species existence in habitats with frequent drops in oxygen. In the laboratory, *Spalax* can survive 3% O_2_ levels, which makes it one of the most hypoxia-tolerant animals known [3, 4]. The extraordinary hypoxia tolerance of *Spalax* is achieved through several morphological and physiological mechanisms of respiration-related genes and their regulation [3–5] including high blood vessel density, which results in a shorter diffusion distance for oxygen and high levels of activity compared to other murine species [3–5].

The mitochondria’s increased production of reactive oxygen species has been observed [6] under hypoxia/reoxygenation cycles, which in turn leads to oxidative stress and damage to the DNA. Because hypoxia also causes dNTP depletion and the repression of DNA repair pathways [5] *Spalax* has fine-tuned various hypoxia-tolerance strategies during its life history, which seems to have driven a large degree of resistance against cancer development in order to allow survival and fitness in its subterranean habitat. Indeed, spontaneous cancer has never been observed during decades of research on *Spalax*. A key mechanism that contributes to cancer prevention is the high repair capacity constantly high expression of DNA repair and editing machinery, especially genes associated with the Fanconi anemia DNA repair pathway in *Spalax* [5, 7, 8]. Another notable mechanism is the heparanase alternatively spliced variant, which inhibits extracellular matrix degradation, tumor growth, and metastasis [9].

A previous study has reported that *Spalax* individuals are predominantly resistant to carcinogenic compound treatment and that fibroblasts play a prominent role in this resistance [10] In that study, *Spalax* fibroblasts were able to inhibit growth and kill adjacent cancer cells of humans and other mammals via direct cell-to-cell interaction in co-culture models, or via fibroblast-generated conditioned medium when transferred to cancer cells growing alone. This scenario was accompanied by decreased cancer cell viability, disturbed cell cycle progression, nuclei deformation, and mitochondrial fragmentation.

In the current investigation, we have attempted to elucidate some of *Spalax*’s molecular mechanisms that enable cancer resistance at the gene-regulation level. We compared the response of *Spalax* and mice to a single subcutaneous injection of the chemical carcinogen 3-methylcholanthrene (3MCA) to the left flank of the neck. All 12 injected mice developed the expected fibrosarcoma tumor 10 to 14 weeks following the injection. Out of the 22 injected *Spalax* individuals, however, two animals developed a benign granuloma after 14 and 16 months, and only two developed the expected fibrosarcoma 18 months [10] and 30 months (reported here) after the injection.

By comparing the transcriptome of *Spalax*’s and mice’s injected versus uninjected tissues and untreated samples, we unraveled differences in the regulation of some of the major genes and pathways that seem to contribute to *Spalax*’s significantly high cancer resistance. We found a higher elevation of anti-cancer pathways in *Spalax* and that many of the differentially regulated genes belong to three major groups: the innate immune system, the extracellular matrix (ECM), and DNA repair machinery. We have concluded that the stronger activity of the *Spalax*’s innate immune system and the elevated expression of tumor suppressor genes related to the ECM compared to those in mice, may play an important role in *Spalax* tumor surveillance. These regulations, together with the *Spalax*’s basic higher levels of expression of genes that belong to DNA damage repair pathways and DNA metabolism, may enable *Spalax*’s higher tolerance to carcinogens (in particular 3MCA) and to its generally extraordinary cancer resistance.

## Results

**Fig. 1:**
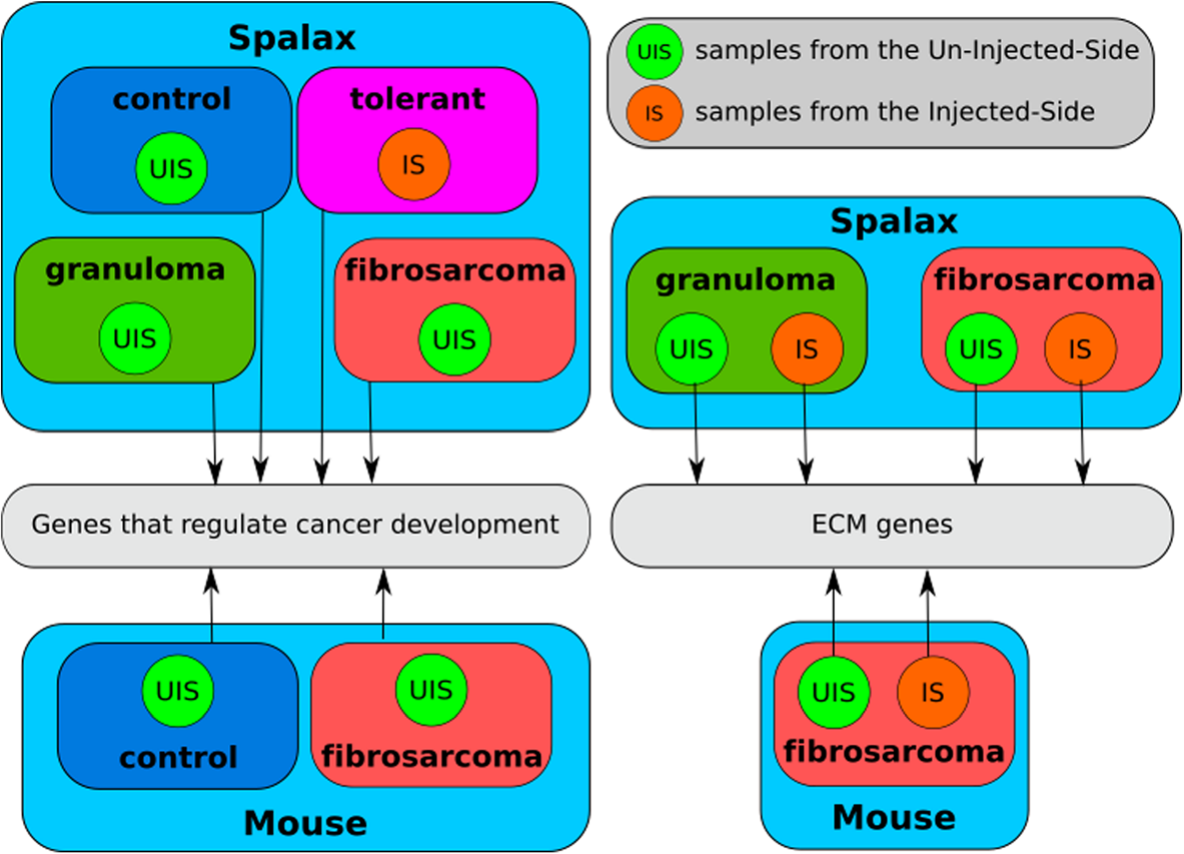
Data sets used in the specific experiments. *Diagram depicting the data usage of the experiments*

*Spalax* and mice were treated with the carcinogen 3MCA; muscle tissue samples were then collected from the injection site, the developed tumor, the opposite (uninjected) side, or the untreated control (Details shown in Table 1). Since we had samples from various conditions, we tried to organize the investigation in the following fashion. To uncover a possible genetic predisposition to cancer resistance and cancer influence on the surrounding tissue, we compared tissue from untreated *Spalax* to tissue taken from the uninjected side of the neck of *Spalax* with tumor growth. We extended this comparison to tissue from mice (which we consider tumor susceptible) to identify genes related to universal cancer development (Fig. 1).

**Table 1:** List of animals used in this study

Species	ID	Name	Sex	Treated	Diagnosis	# Samples		Time of operation
						UIS	IS	
*Spalax*	1042	*Spalax* control	F	–	healthy	1x	–	–
*Spalax*	1075	*Spalax* control	F	–	healthy	1x	–	–
*Spalax*	2095	*Spalax* control	F	–	healthy	1x	–	–
*Spalax*	6038	*Spalax* control	F	–	healthy	1x	–	–
*Spalax*	2251	*Spalax* tolerant	M	3MCA	healthy	–	1x	18 months
*Spalax*	2261	*Spalax* tolerant	M	3MCA	healthy	–	1x	18 months
*Spalax*	2218	*Spalax* tolerant	M	3MCA	healthy	–	1x	30 months
*Spalax*	2375	*Spalax* tolerant	F	3MCA	healthy	–	1x	30 months
*Spalax*	600	*Spalax* granuloma	F	3MCA	granuloma	1x	1x	14 months
*Spalax*	976	*Spalax* granuloma	F	3MCA	granuloma	1x	1x	16 months
*Spalax*	2240	*Spalax* fibrosarcoma	F	3MCA	fibrosarcoma	1x	1x	18 months
*Spalax*	2230	*Spalax* fibrosarcoma	M	3MCA	fibrosarcoma	1x	1x	30 months
mice	M1	mice control	M	–	healthy	1x	–	–
mice	M2	mice control	M	–	healthy	1x	–	–
mice	M5	mice control	M	–	healthy	1x	–	–
mice	1019	mice	M	3MCA	fibrosarcoma	1x	1x	14 weeks
mice	1023	mice	M	3MCA	fibrosarcoma	1x	1x	12 weeks
mice	512	mice	M	3MCA	fibrosarcoma	1x	1x	11 weeks

Outlining the animals used in this study. The number of samples is separated into the uninjected side and injected side. The time of operation is the time after treatment with 3MCA

In an attempt to uncover cancer-resistance mechanisms and to further understand what makes *Spalax* generally different in its non-susceptibility to cancer induction, we compared samples from tumor-resistant *Spalax* with samples retrieved from four *Spalax* individuals that failed to resist chemical carcinogenesis to different degrees.

### Identification of genes expressed in carcinogen-tolerant *Spalax*

We took samples from various conditions in *Spalax* and mice (Table 1). Four samples of untreated *Spalax* animals as the control group were used, as well as four samples from animals that were injected but did not develop tumors after 18 and 30 months, which we term “*Spalax* tolerant.” Out of 22 treated *Spalax*, four developed a tumor: two a malignant fibrosarcoma and two a benign granuloma. The granuloma samples were pathologically defined as spindle cell proliferation reflecting fibrosis at the site of an incompletely resolved inflammation, whereas fibrosarcomas were described as highly mitotic and heavily inflamed spindle and epithelioid cell tumors [10]. From these animals, samples were taken from the injected side, or IS (i.e., from the tumor), and from the uninjected side (UIS) of the neck. All treated mice developed a tumor. Samples were also taken from three mice on the IS with the tumor and the UIS. In addition, samples from three untreated control mice were used as control.

**Fig. 2:**
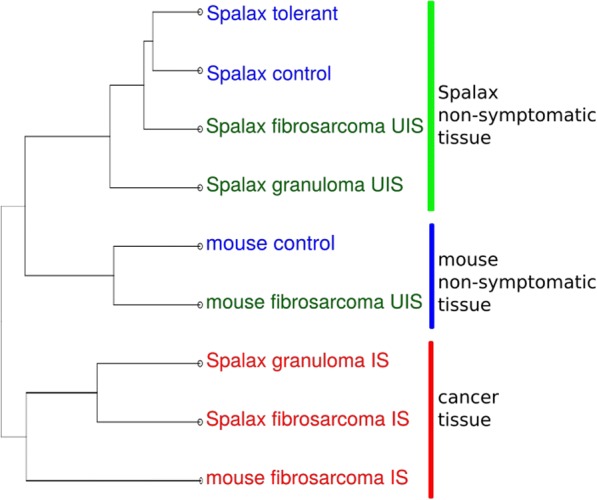
Clustering of the logarithmized expression values. The samples of *Spalax* with no tumor growth group are in one clade, accompanied by samples without symptoms. The non-symptomatic mouse samples were grouped together as well. All samples that were taken from tumor growth are shown in red

After filtering, trimming, and mapping these RNA-Seq samples, we investigated the effects of the 3MCA treatment and the relation between these samples. We then clustered the expression values (Fig. 2). The analysis showed a separation of the samples by phenotype first, and then by species. The samples of *Spalax* untreated control and “*Spalax* tolerant” were direct neighbors. Close to this subgroup were the UIS samples of *Spalax* with fibrosarcoma and then granuloma. Mice control and mice UIS samples were also paired together but were still closer to the non-carcinogenic tissues of *Spalax* than to the carcinogenic tissue samples. All samples taken from tumor tissue were clustered in the same branch, first pairing samples from *Spalax* and then mice.

Because the development of tumors has an extensive impact on gene expression, it is generally difficult to distinguish between expression changes that allow tumor growth from those that are subsequently induced by the tumor. To assess how cancer development in *Spalax* affects genome expression in the neighboring tissues, we compared the *Spalax* control with the *Spalax* UIS from the animals with tumors by pooling them together. We discovered 68 annotated genes to be differentially expressed (Additional file 1: Table S2). To determine which of these 68 genes could be considered as plausible candidates for cancer susceptibility, we added mice samples to the analysis. Mice have a very low cancer resistance, so we were interested in the expression behavior of these genes in mice samples. We clustered the 68 genes according to their expression patterns and included all data sets at our disposal, excluding tumor tissue, namely *Spalax* UIS (of granuloma and fibrosarcoma bearing animals), mice control, and mice UIS (fibrosarcoma bearing). In this part of the study we omitted the tumor tissue to avoid introducing the large variances found in tumors.

**Fig. 3:**
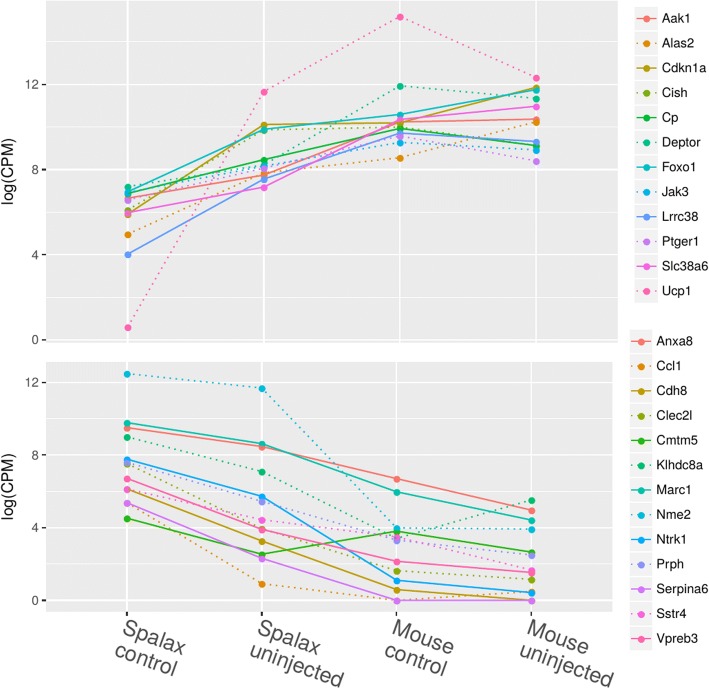
Genes that show the same pattern in *Spalax* control vs. all other samples. The Spalax uninjected pools sample includes both Spalax with fibrosarcoma and granuloma. While Spalax tolerant resisted the 3MCA injection, Spalax uninjected, mouse control, and mouse uninjected are considered “susceptible” to tumor growth. The upper diagram shows genes that are generally up-regulated in all susceptible samples; the lower diagram shows genes that are generally down-regulated

In this clustering, we found 25 genes with distinct expression patterns (Fig. 3). Twelve of these genes displayed a general up-regulation in the susceptible samples compared to the *Spalax* control. These genes contain known cancer-related genes such as *Jak3*, *Foxo1*, and *Cdkn1a* (p21). The remaining 13 genes showed a general down-regulation in the susceptible tissue samples, i.e., of mice and tumor-bearing *Spalax*. We found known cancer-related genes such as *Ntrk1*, *Serpina6*, *Nme3*, and *Sstr4*. We also found significant differences in the level of expression of mitochondrial uncoupling protein (UCP1) in tumor-resistant samples (*Spalax* control and *Spalax* UIS cancer negative) in comparison with the cancer-sensitive group (mouse control, mouse UIS, and *Spalax* UIS cancer positive). The low level of UCP1 expression in the tolerant samples may have reflected a high level of natural defenses against cancer via mitochondrial homeostasis. Nucleoside diphosphate alkylase 2 (NME2), which is an active cancer suppressor, was highly expressed in *Spalax* tissues (Fig. 3).

**Fig. 4:**
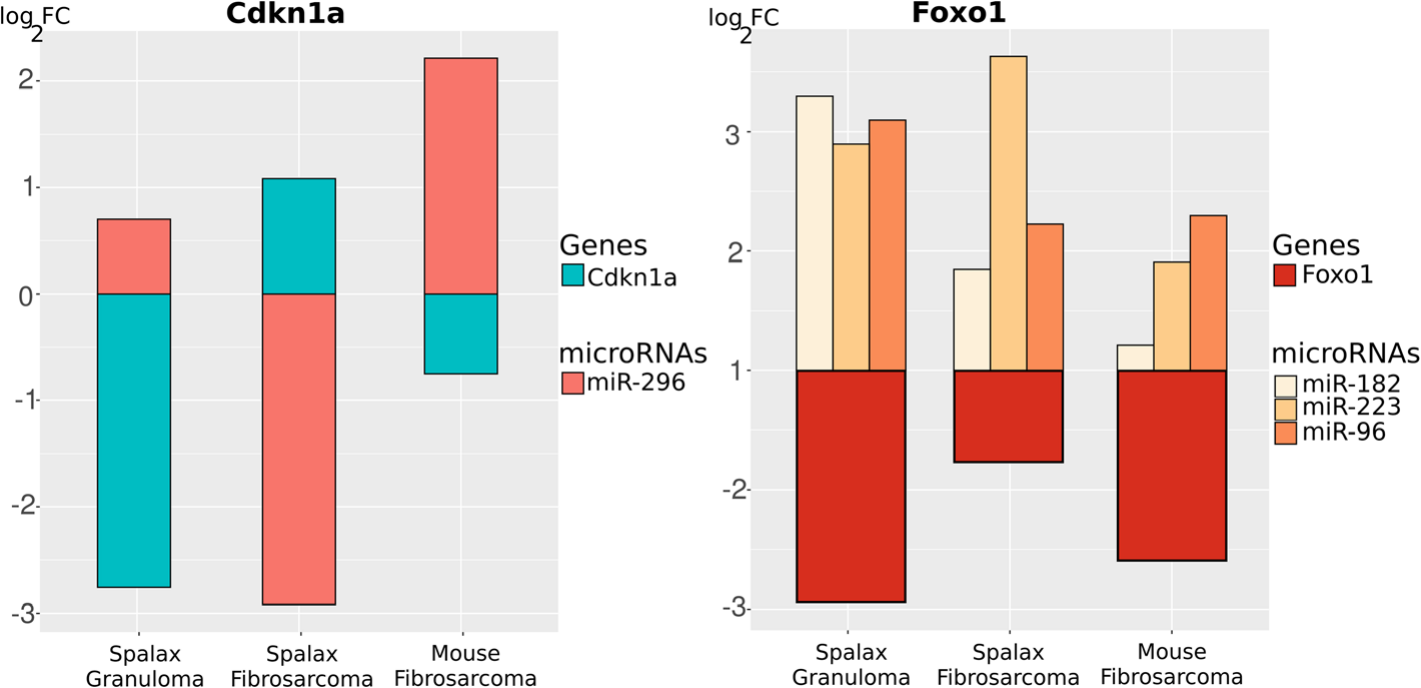
Expression patterns of Cdkn1a and Foxo1 and their regulating microRNAs. Since microRNAs silence genes, the expression of the genes and their microRNAs is reversed

We then conducted microRNA analysis in *Spalax* and mice tumor-bearing individuals. microRNAs are small (~ 22 nucleotides) non-coding RNA molecules. They act as post-transcriptional modifiers that can silence a gene. We found nine microRNAs that were connected to the candidate genes, either as microRNA regulators or as potential targets. Differential expression analysis was performed for the comparison of UIS vs. IS in granulomas and fibrosarcomas in *Spalax* as well as for fibrosarcomas in mice (Additional file 1: Table S3). We found 27 microRNAs that targeted *Cdkn1a* and *Foxo1*. Since microRNAs act as suppressors of gene expression, we expected the microRNAs and the target genes to have opposing expression patterns in the three comparisons between the UIS and IS in *Spalax* granuloma, *Spalax* fibrosarcoma, and mice fibrosarcoma. Three microRNAs that target *Foxo1* showed this pattern (miR-182, miR-223, and miR-96), and one for *Cdkn1a* (miR-296), as shown in Fig. 4. Although which genes regulate these microRNAs remain unknown to date, we hypothesize this pattern as a partial explanation for the regulation we observed.

### Investigation of *Spalax* individuals that developed cancer

Since cancer development is a relatively rare event in *Spalax*, we were especially interested in investigating the four animals that did develop tumors, specifically the transcriptome differences between those that managed to limit the growth to a benign granuloma and those that formed a malignant fibrosarcoma, similarly to mice. To explore the differences between the two groups, we compared the uninjected side of the treated *Spalax* with the injected, tumor side of the animals using gene expression analysis. In the comparison of the samples of granuloma tissues, we found 2316 differentially expressed genes (DEGs). The fibrosarcoma samples showed 3293 DEGs. The intersection of these two gene sets consisted of 1736 genes. We split the functional analysis into three parts:

1. genes that were differentially expressed in both tumor types (Additional file 1: Table S7);
2. genes that were differentially expressed only in animals with granulomas (Additional file 1: Table S8); and
3. genes that were differentially expressed only in animals with fibrosarcomas (Additional file 1: Table S9).

Functional analysis of the DEGs showed that in all three comparisons, the dominant categories were the *extracellular matrix*, the *citrate cycle*, the *cell cycle*, and the *immune system*. These categories are all typically influenced by cancer development. We also directly compared the tissue from the uninjected sides of *Spalax* with fibrosarcoma and *Spalax* with granuloma (section 3.3). We found only 84 DEGs, indicating that despite the different tumors, the tissues were very similar. Once again, ECM-related categories dominated the enrichment analysis.

Since several of the tests showed an enrichment of genes belonging to the extracellular matrix and the immune system, we focused our investigation accordingly.

We focused on genes that were related to the extracellular matrix and collagen formation, as they were found to be significantly differentially expressed in both fibrosarcoma and granuloma tumors. This analysis showed that genes were generally up-regulated in the ECM categories (Additional file 1: Table S1). This finding is not surprising, since the modification of the extracellular matrix is a hallmark of cancer that allows growth and invasion into neighboring tissue and stimulates blood vessel growth. We continued to investigate the ECM-related genes on their connectivity, stability in expression (i.e., low variance between the samples of the same condition), and the known effects on tumor growth. We found a highly connected network of seven genes, namely *Aspn*, *Chad*, *Fbln, Kera, Lum, Omd*, and *Sfrp1.* (See Table 2 for a description of these genes.) The genes are connected via shared protein domains and co-expression, which were found using GeneMANIA annotation software [11] and various publications [12–14].

**Table 2:** Short description of the ECM genes that form a regulatory network

*Aspn*	(Asporin) is an extracellular matrix protein that modulates the Transforming Growth Factor β (TGFβ) signaling pathway, regulating cartilage matrix gene expression and cartilage formation [60]. *Aspn* inhibits TGFβ1, a biomarker of poor prognosis in cancer [61]. High asporin expression is significantly associated with less aggressive tumors, which could explain the benign growth.
*Chad*	(Chondroadherin) expression has been linked to significant decreases in hepatocellular carcinoma, both in mRNA and protein levels. *Chad* abundance correlates with differentiation and metastasis, while the reduction of *Chad* levels significantly enhances proliferation and migration [39]. *Chad* has a α2β1 integrin binding sequence that previous studies have found counter cancer development [62].
*Fbln5*	(Fibulin-5) is frequently silenced in lung cancer and suppresses cell invasion by inhibiting the *Wnt*/β-catenin pathway [63]. *Fbln5* has also been shown to reduce reactive oxygen species (ROS) production by modulating cell-ECM interactions [64]. Excessive ROS production results in cellular toxicity, which can promote tumor growth.
*Lum*	(Lumican) has different effects on cancer development, depending on the type of tumor and whether *Lum* is expressed in the cell or in the adjacent stromal tissue [65]. *Lum* also acts as an oncogene, but if present in the surrounding stroma, it can restrict cancer progression and migration.
*Sfrp1*	(Secreted frizzled-related protein 1) is an antagonist of *Wnt* signaling, which gives *Sfrp1* its tumor-suppressive effect. The loss of *Sfrp1* has been hypothesized to activate MAPK or the non-canonical *Wnt* pathway [66]. One mechanism of how this loss can occur is the methylation of *Lum* via HP1α.
*Kera*	(Keratocan) encodes a protein that is involved in corneal transparency. Mutations can cause cornea plana [67].
*Omd*	(Osteomodulin) is an extracellular matrix keratan sulfate proteoglycan that is also connected to bone development [68].
*Igf2*	(Insulin-like growth factor 2) is a mitogenic peptide hormone expressed by liver and many other tissues. It is closely associated with cancer [69, 70].
*Cebpa*	(Transcription factor CCAAT / enhancer-binding protein alpha) is an important protein during embryogenesis, glucose metabolism, adipogenesis, and myeloid development [16, 30, 71].
*Jun*	*Jun* is part of the transcription factor activator protein 1 and has been closely associated with cancer development [15, 72, 73].

**Fig. 5:**
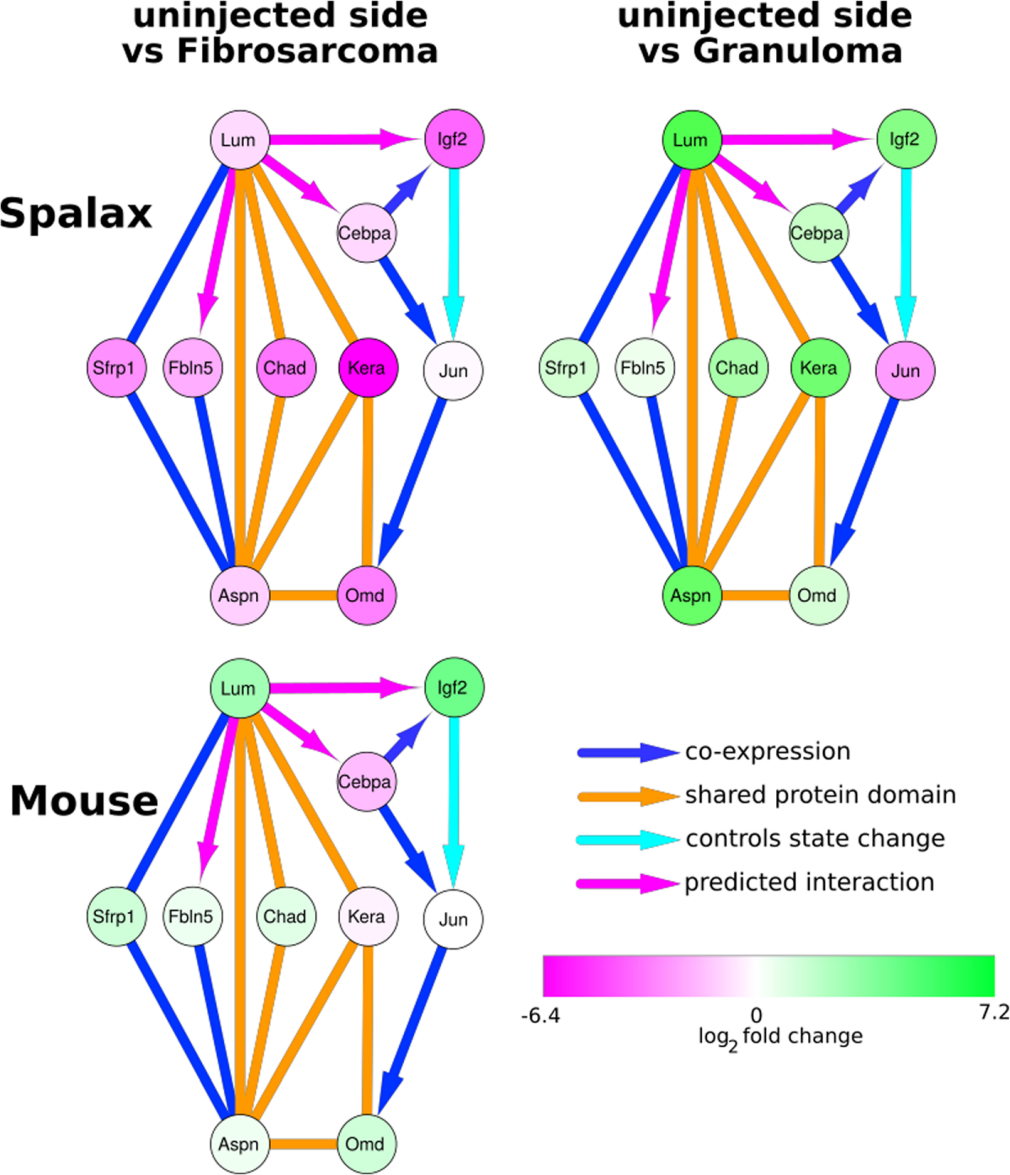
Regulatory center genes in different animals and conditions. The color of the nodes represents the log2 fold change in the comparisons of the uninjected with the injected tissue

Figure 5 shows the connected genes, where *Lum* and *Aspn* are highly connected hub genes. Since it is difficult to determine the regulatory direction of co-expression, and a shared protein domain is by its nature undirected, it is also difficult to distinguish between regulators and targets. Our analysis showed that this gene complex was generally up-regulated in *Spalax* granulomas and mice fibrosarcomas (compared to uninjected tissue) while being down-regulated in *Spalax* fibrosarcomas.

In addition to the cellular matrix genes, we also found connections to the cell growth genes *Igf2*, *Jun*, and *Cebpa*. While they are not directly involved in matrix generation, they are connected to the central ECM gene *Lum*. *Jun* is part of the hetero-dimer *AP-1*, and centrally involved in prostate cell migration and invasion. Furthermore, *AP-1* was reported having a significant role in melanomas [15]. *Cebpa* is *down-regulated* in gastric cancer, and potentially useful for its treatment [16]. Nevertheless, *Igf2* and *Cebpa* were clearly regulated differently in *Spalax* fibrosarcoma samples than in the granuloma samples.

In mice, the picture was more ambiguous. While the genes related to cellular matrix complex are generally up-regulated, the intensity of the up-regulation is much smaller, and often not statistically significant.

While not presented in this network, other important ECM-related genes were also found to be differentially regulated in *Spalax* and mice. The gene *Creb3l1* was found to be more highly expressed in *Spalax* granulomas compared to the fibrosarcomas of both mice and *Spalax*. In addition, *Emilin2* is up-regulated and inhibits the *Wnt*-signaling pathway [17]. Other genes were also found to be up-regulated, including *Bmp7*, which antagonizes transforming growth factor β1 (TGFβ1)-mediated fibrosis through suppressing epithelial-mesenchymal transition [18], and *Mmp8*, which prevents metastasis formation through the modulation of tumor cell adhesion and invasion [19].

We then performed microRNA analysis of the ECM genes. We used the microRNA data to explain the impact of the genes involved. We found nine microRNAs that were connected to the ECM genes, either as micoRNA regulators or as potential targets. Differential expression analysis was carried out for the comparison of UIS vs. IS in granulomas and fibrosarcomas in *Spalax*, and in fibrosarcomas in mice (Additional file 1: Table S4). Four of these nine microRNAs were found to be regulated by *Cebpa*, yet none matched the expression pattern of *Cebpa* as a gene. We believe that an additional regulatory mechanism must be responsible for this expression pattern. We must point out, that the knowledge about microRNAs and genes were taken from mice annotation. This has to be taken into consideration when evaluating the impact of these findings.

In this part of the study, we compared the two different tumor tissues (granuloma and fibrosarcoma) to uninjected tissue. We identified a tight network of genes from the extracellular matrix, which then allowed us to hypothesize that the modulation of ECM in *Spalax* granuloma largely contributes to host resistance.

### Direct comparison of uninjected tissue of *Spalax* with tumor

In general, the transformation from normal cell to cancer cell is accompanied by an extensive altering of gene expression. In the current experimental setup, it was difficult to determine which changes in the observed major shift in gene expression were the drivers of cancer development and which changes were secondary to tumorigenesis. This situation is especially important in order to understand the difference between *Spalax* and mice. We started by comparing the uninjected side (UIS) with the injected side (IS) in *Spalax* to determine the extent of genetic rewiring. We considered a |log2FC| ≥ 2 as a threshold for highly differentially expressed genes (see Additional file 1: Table S6). Among all DEGs in the described experiments, we filtered the genes that belong to these categories: cell cycle, citrate cycle, immune system, extracellular matrix, hypoxia response, and degenerative diseases (Table 3). The ECM has the most differentially expressed genes of these categories, followed by cell cycle.

**Table 3:** Analysis of the highly differentially expressed genes following 3MCA injection

	Immune	ECM	Hypoxia	Cell cycle	Citrate cycle	Degenerative diseases	# of genes
HDEG^a^	125	269	164	239	74	159	888
DE^b^	43	57	88	84	9	21	220
TSU^c^ S > M^d^	13	14	8	6	1	5	39
TSU M > S^d^	4	4	3	5	–	5	21
TPD^e^ S < M^f^	4	7	14	9	–	4	36
TPD M < S^f^	4	9	6	6	–	5	27

^a^*HDEG* highly differentially expressed genes |log2f| ≥2

^b^*DE* Differentially Expressed—the number of HDEG refers to when the difference in the change between the species following the 3-MCA injection is |log2f| ≥1

^c^*TSU* Tumor Suppressors Up-regulated

^d^S > M, M > S: higher elevation in the *Spalax* compared to the mice, or the mice compared to the *Spalax*, respectively

^e^*TPD* Tumor Promoters Down-regulated

^f^S < M, M < S: stronger down-regulation in the *Spalax* compared to the mice, or the mice compared to *Spalax*, respectively

To investigate the differences between the species, we included the mice tumor samples in our analysis. We looked at genes that showed an expression difference between the species of |log2fc| ≥ 1. The highest differences were found in hypoxia-related genes (88/164 genes, 54% of the group members). The second group was related to the cell cycle control (84/239, 35% of the group members), the third was the immune system group (43/125 genes, 34%), and the fourth was the ECM group (57/269, 21%).

In the analysis, *Spalax* showed a larger number of up-regulated tumor suppressors (39:21) and more down-regulated tumor promoters compared to mice (36:27). Most of the anti-cancer reprogramming of gene expression in *Spalax* seems to occur in the three gene groups of immune system 17/43 (40%), ECM 21/57 (37%), and hypoxia responsive 22/88 (25%).

The elevation of *Tlr4*, *Ifngr1*, *Ifnar2*, *Stat1*, and *Irf1* is presumably a part of an integrated pathway. The levels of *Stat1* in *Spalax* in response to 3MCA injection were found to be five times higher compared to mice. These elevated *Stat1* levels seemed to contribute to the tumor surveillance Interferons-I, which were found to systemically activate natural killer cell activity.

We also found stronger down-regulation of *mTOR* mRNA expression in *Spalax* compared to mice. In this regard, the elevation of the hypoxia-responsive *Ddit4* in *Spalax*, compared to its down-regulation in mice, should also be noted, as up-regulation of *Ddit4* expression mediates *mTOR* inhibition and growth inhibition in cancer cells [20].

**Fig. 6:**
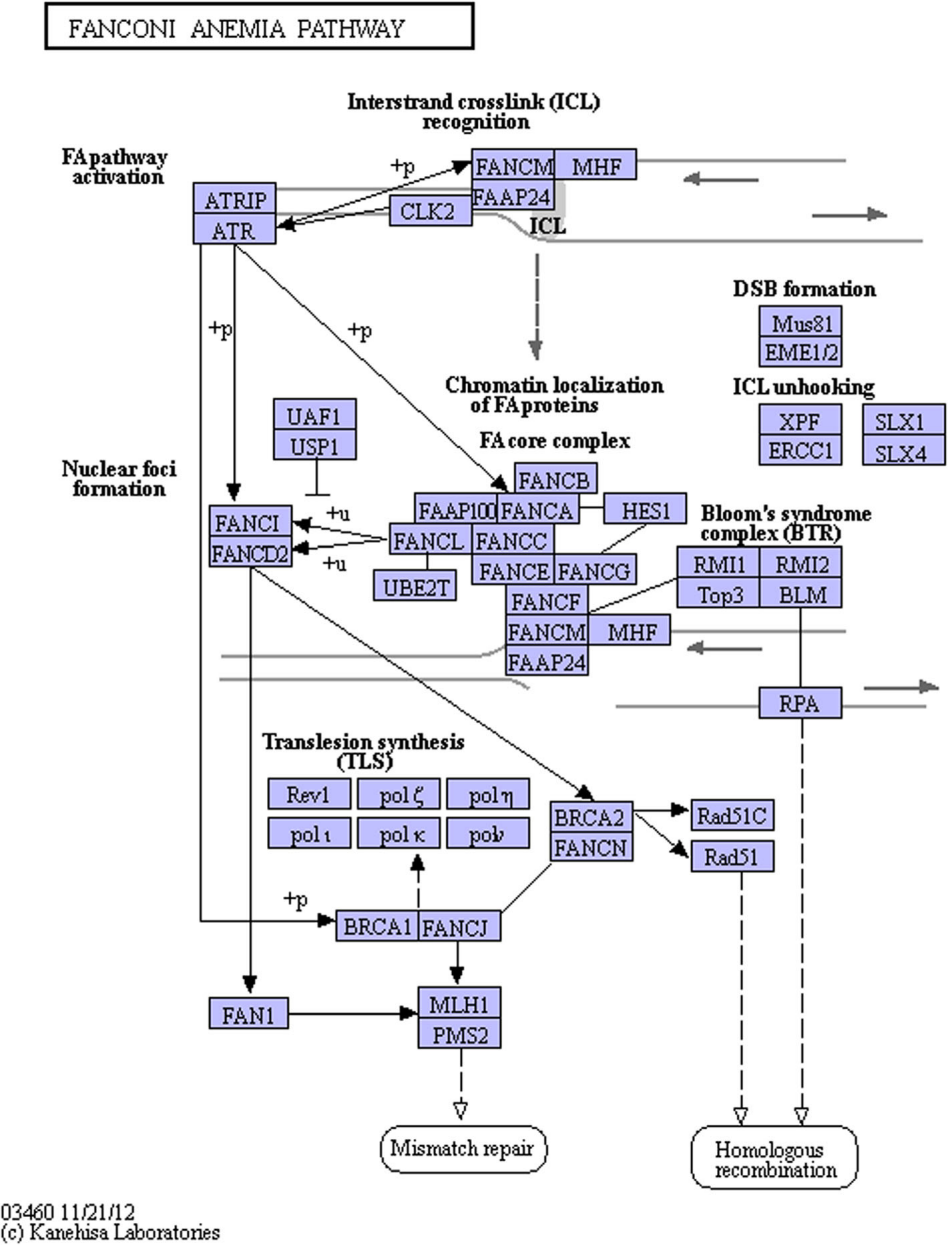
The Fanconi Anemia pathway

By comparing the control, untreated *Spalax*, and mice groups, we found higher basic levels of expression of 75% of the 54 genes that belong to the Fanconi Anemia pathway. Fanconi Anemia genes are involved in the homologous recombinational repair of DNA double-strand damage, inter-strand crosslinks repair, and mismatch repair. The ratios between the control groups of the mRNA expression (*Spalax*/mice) were found to be high for most of the genes (Table 4 and Fig. 6).

**Table 4:** List of Fanconi anemia genes

Gene	up regulation
Atrip	60x
Brca1	3x
Brca2	10x
Cenps	3x
Eme1	× 336
Fanca	10x
Fancc	10x
Fanci	55x
Fancn	55x
Fen1	178x
Pms2	5x
Rad51c	11x
Rmi2	6x
Top3	5x
Wrn	3x
Telo2	5x

List of Fanconi anemia genes that are up-regulated between *Spalax* control and mice control

## Discussion

The treatment with 3MCA resulted in the development of fibrosarcoma in 12 out of 12 mice within 3 months, while only 4 out of 22 *Spalax* developed tumors during this time; two of them developed a benign granuloma by 14 and 16 months, and two had developed the expected fibrosarcoma by 18 and 30 months following the carcinogenic treatment. For malignant tumors only, Fisher’s exact testing showed significant differences (*p*-values of 3.32 × 10^−6^ and 1.66 × 10^− 7^ for all tumor outcomes).

We showed in this study that *Spalax* is highly resistant to the carcinogenic effects of 3MCA injection. While the investigation of “confirmed” cancer resistance is important for the understanding of cancer mechanisms, the investigation of failed cancer resistance is no less valuable. Of special interest in this study is the fact that four *Spalax* failed to escape tumor growth in different ways, where two of them managed to “contain” the process, which ended in the formation of benign tumors. Although two cases of each type were the smallest sample size for statistics purposes, this examination provided an opportunity to gain deep insights into the carcinogenesis of these subterranean mammals.

The first investigation of the samples via clustering showed that the most significant division between the samples was between tumor tissue and non-treated tissue, with the genus as the second divider (Fig. 2). Since tumors are generally considered to be organs developing in the context of organisms [21], tumor growth has a larger impact on the overall expression than the species factor. The *Spalax* tolerant samples clustered together with the *Spalax* control, which shows that the *Spalax* that did not develop a tumor were indeed affected very little by the treatment. The UISs of the fibrosarcoma and granuloma individuals were positioned on neighboring branches of the graph, showing that the tumor did seem to have a major impact on genome expression in the cells of neighboring tissue. Interestingly, the UISs of the fibrosarcoma tissue was closer to the *Spalax* control/tolerant than the UISs of the granuloma tissue, which was also visible during other parts of the investigation. We hypothesize that *Spalax* with benign tumors mounted substantial defense mechanisms, which were reflected in the extensive changes in gene expression in the neighboring tissue.

We found that the mice control and mice UIS of fibrosarcoma had their own branches and were still closer to the untreated samples of *Spalax* than to the clade with all the tumor tissues, which implies that extensive rewiring in the gene expression caused by the tumor growth occurred. We found 25 genes that behaved consistently different in animals that developed any type of tumor regardless of the species (Fig. 3). While some of these genes have been described in the literature as being related to cancer, our work can extend that knowledge by showing how these genes react in different species and conditions. Some of these genes are known cancer-promoting or suppressing genes such as *Cdkn1a* and *Foxo1*. While the precise mechanisms are unknown, and extensive investigations will be required to validate these findings, we propose these genes/gene networks as candidate genes that contribute to cancer resistance in *Spalax*.

Since the population of *Spalax* is not homogeneous, like any population of non-inbred wild animals, it is natural to assume that among the heterogeneous group will be those that cannot avoid the carcinogenic stimulus. On the contrary, the cancer-prone population of the laboratory mice demonstrated rapid response to carcinogen treatment by the development of fibrosarcoma. Thus, tissues for analysis from untreated and UIS treated mice were considered to be samples with very low threshold levels. To determine whether individual tumor sensitivity in *Spalax* is genetically pre-determined, we organized the samples for comparative analysis as follows [1]: cancer sensitive (mouse untreated and UIS of tumor-bearing mice as well as UIS of *Spalax*, in which we observed the neoplastic processes) and [2] cancer resistant (*Spalax* control plus *Spalax* tumor-negative UIS). See Fig. 3.

During tumor progression, already mutated cells constantly co-interact with stromal host cells, which can either promote or suppress the growth of malignant cells [22]. In this context, the outcome depends to a large extent on the abilities of the stromal host cells to maintain homeostasis, integrity, and oxidative metabolism. According to the paradigm of tumor cell metabolic parasitism, host cells “fuel” the anabolic growth of cancer cells through energy transfer [23].

The role of the mitochondrial metabolism in the development of cancer is of great interest. Mitochondrial uncoupling protein 1, or UCP 1 (also known as thermogenin), was originally associated with the brown adipose tissue of mammals. UCP1 promotes proton leakage from the inner mitochondrial membrane independent of ADP phosphorylation; this leakage uncouples respiration from ATP synthesis, thus dispersing the energy of oxidation in the form of heat [24, 25]. Previous studies have demonstrated the unique changes in the *UCP1* sequences of the naked mole rat and have discussed these sequences’ possible roles in these mammals’ thermoregulation and longevity [26]. UCPs are currently being investigated for their potential role in cancer initiation and development [27]. In studying the role of UCP1-overexpressing cancer-associated fibroblasts (hTERT-BJ1) in human breast cancer cell (MDA-MB-231) growth, a previous study has demonstrated increased β-oxidation, ketone body production, and the release of ATP-rich vesicles, all of which “fuel” tumor growth [23]. Conversely, the induction of mitochondrial dysfunction via UCP transfection of MDA-MB-231 cells results in significant reductions in tumor growth. Thus, if a rapidly growing tumor does not receive “fuel” from surrounding tissues, then the tumor may undergo involution by “eating itself.” The comparative analysis of cancer-sensitive and cancer-resistant samples in our study revealed a dramatic difference in the levels of *UCP1* gene expression (Fig. 3). We can assume that a high level of expression of *UCP* in tissues may represent a predictive marker for carcinogenesis susceptibility.

Another prognostic marker of cancer development, nucleoside diphosphate alkylase 2 (*NME2*), was differentially expressed in our study’s mouse samples (low expression level) and in *Spalax* samples (high expression level), including samples derived from the UIS of cancer-sensitive *Spalax* (Fig. 3). *NME2* have been extensively studied in the past for their cancer-suppressing activities [28]. Previous studies have shown that an overexpression of NME2 reduces the migration and invasion of gastric cancer cells to the cellular matrix in vivo and in vitro. Subsequently, NME2 expression is associated with the well-differentiated and less invasive histology of gastric cancer. The high level of expression of NME2 gene in the *Spalax*-sensitive samples found in our study may explain to a certain extent the significant delay of tumor development and the tumors’ benign nature in half the cases. In addition, Somatostatin Receptor 4 (*SSTR4*), which we found to be more highly expressed in *Spalax* tissues (Fig. 3), could inhibit the proliferation of normal cells or tumor cells and suppress the formation of tumor vessels via releasing hormone-inhibiting tumor growth [29].

Even though four *Spalax* animals failed to resist the treatment with 3MCA in our study, this failure occurred in two distinct ways. We explored the differences between the benign granuloma and the malignant fibrosarcoma. One of our main findings was a cluster of key regulators in the extracellular matrix pathway. The ECM gene cluster including the *Lum, Sfrp1, Fbln5, Chad, Kera, Aspn*, and *Omd* genes was found to be distinctly more activated in granulomas than in fibrosarcomas. Especially noteworthy is that the activation of highly connected tumor suppressors and anti-metastatic genes such as *Lum* and *Aspn* may have explained the stunted growth of the tumor. The cell growth genes *Igf2* and *Cebpa* are also up-regulated in granuloma. These genes are generally up-regulated in cancer cells, although *Cebpa* has also been reported to act as a tumor suppressor [30]. It was interesting to note that these cancer genes were activated in the benign tumor in our study, while the malignant growth seemed to have deactivated these genes..

The extracellular matrix plays a significant role in cancer, with both tumor promoting and inhibiting effects [31]. The switch in the citrate cycle is likely related to the Warburg effect, where the tumor switches from aerobic to anaerobic glycolysis. This switch enables the tumor to use the available glucose for other processes, such as cell division [32]. Removing cell cycle checkpoints is a basic requirement for the uninhibited proliferation of tumor cells, while cell cycle arrest is a common mechanism involved in tumor suppression [33]. Regulating the genes of the immune system is a crucial factor to prevent the organism from attacking the tumor [34].

*Creb3l1* is an endoplasmic reticulum gene that is a transcription factor and a metastasis suppressor. It has been found to repress the expression of the genes that regulate metastasis, invasion, and angiogenesis in both breast cancer and bladder cancer [35]. We found in our study that the expression of *Creb3l1* was higher in the granulomas of *Spalax* compared to the fibrosarcomas and may have been an important factor in preventing the development of malignant fibrosarcoma.

We attempted to elucidate the connections between these ECM genes in our study. As shown in Figs *5*, 13 interactions were found to belong to the class of shared protein domain or co-expression. Because the nature of these interactions makes it impossible to infer a direction of the regulation, it is difficult to determine which gene induced a reaction and which genes were influenced. We did find a few differentially expressed microRNAs, mainly regulated by *Cebpa*. The expression patterns of the microRNAs and *Cebpa* did not match, however, thus indicating the presence of complex regulation activities and additional interactions that we are not aware of. Another explanation is, that there are species specific microRNAs, that are not present in mice.

The hypoxic environment *Spalax* lives in causes DNA damage through the occurrence of reactive oxygen species production. *Spalax* has adapted to this over the years by developing a very efficient DNA repair mechanism [36] that subsequently prevents persistent DNA damage and ultimately prevents cancer development by repairing harmful mutations. 3MCA is a DNA-damaging agent, and *Spalax* reacts to this damage by activating DNA-repair genes, especially the Fanconi anemia genes. In our study, most of the Fanconi anemia genes already had a higher expression in the untreated control groups. Similar results have been shown in a previous comparison between the transcriptome of the brain and liver [5, 7] (Additional file 1: Table S5).

In addition to its enhancement of the DNA-repair effect, the hypoxic habitat of *Spalax* also appears to make the expression of hypoxia-related genes more attuned to countering cancer development. We found that more tumor suppressors up-regulated and more tumor promoters down-regulated in *Spalax* compared to mice in the hypoxia-responsive gene group. In this regard, one of the most important hypoxia-responsive genes is *mTOR*, which is a master regulator of cell growth control [37]. Previous studies have implicated downstream *mTOR*-regulated processes in the hallmarks of cancer: proliferative signaling, metabolic reprogramming, angiogenesis, and metastasis. We suggest that the down-regulated expression of *mTOR* in the hypoxic conditions found in the tumor microenvironment may lead to declines in the metabolic rate and may be an adaptive mechanism that *Spalax* uses to resist cancer development.

Another very important barrier to cancer development is the immune system’s tumor surveillance. In our analysis, compared to mice, *Spalax* displayed a stronger and more robust activation of innate immune response of the tumor suppressors responsible for tumor surveillance. The elevated expression of *Tlr4*, *Ifngr1*, *Ifnar2*, *Stat1*, and *Irf1* that was revealed upon carcinogen treatment appeared to reflect a part of an integrated pathway reaction. Previous studies have shown that these innate immune genes are important players in tumor surveillance [38, 39]. The expression of *Stat1* in our study might have been the major exception because of its higher basal expression in the untreated control group. An earlier study reported that Stat*1* can inhibit the growth of benign and neoplastic cells by regulating the transcription and expression of several pro-apoptotic and anti-proliferative genes [40]. *Irf1* is a transcriptional regulator and tumor suppressor that suppresses tumor cell growth and stimulates an immune response against tumor cells [41]. We believe that these changes in gene expression contribute to the *Spalax-*specific relative tolerance to the carcinogenic 3MCA compared to mice and might be a part of the layer of genes that enable *Spalax*’s high cancer resistance.

As a potential additional defense, we found a stronger up-regulation of the immune system in *Spalax* compared to mice, especially of the innate immune-response tumor suppressors.

One of the major risk factors for cancer is aging. *Spalax* is known for its extreme longevity; the species can reach 20 years old in captivity, which is unusual for a mammal with such a small body mass. Since *Spalax* is also a solitary non-model organism that does not breed in captivity, the exact ages of specimens cannot be precisely determined, which could have been a confounding factor in our analysis. Due to this resistance, only four of the animals in our study developed a tumor, of two different phenotypes: granuloma and fibrosarcoma. These different factors created a high degree of variance in the data, since the samples were taken from only two different animals per tumor type and from animals of different sexes and ages. We addressed this variance in our study by rigorous normalization and filtering of the data.

## Conclusion

We identified 25 genes in this study that we consider important for cancer development. Some of these genes are known to be related to cancer, while the roles of others are still unknown; we propose the latter as potential candidates for further cancer-resistance studies.

This study of the rare cases of failed cancer resistance in *Spalax* showed a tight network of genes that are responsible for the extracellular matrix remodeling. We consider these genes to be important for the difference between malignant and benign tumor development. This study has also pointed out several differences between *Spalax* and the model organism mice. The DNA repair genes of the Fanconi Anemia pathway were found to be especially important factors in cancer resistance.

## Materials & methods

### Animals and tissue extraction

All animal protocols were approved by the University of Haifa Institutional Ethics Committee (Permit# 193/10).The experimental design and the animals tested in the experiments are listed in Table 1. *Spalax* was captured in the field and kept in individual cages in the Animal Facility of the Institute of Evolution, University of Haifa. Mice were purchased from Harlan Laboratories (currently Envigo, Jerusalem, Israel). Twenty-Two *Spalax* and 12 mice (C57BL/6) were treated with 3MCA. All animals were kept at 21°–23 °C in a 12:12 light-dark cycle with free access to food and water. Untreated control *Spalax* animals were anesthetized with isoflurane when biopsies were taken (4% mixed in oxygen for the initial anesthesia; followed by 3% isoflurane flow for 20 min), then animals were kept in the Animal Facility under veterinary observation for recovery. Animals with tumor growth and control mice received 10% isoflurane, continued for more than 2 min after breathing stopped, when the samples were taken.

The 3MCA treatment was done according to Manov et al.’s [10] paper. A single subcutaneous injection of 3MCA was administered to the left-flank of the neck of the *Spalax* and mice. When the tumor length reached about 2–3 cm, it was completely removed. The most proximal ~ 0.5 cm was harvested for RNA extraction, after pathological confirmation defining it as fibrosarcoma. For the control animals or the resistant *Spalax* individuals a similar sized neck muscle biopsy was removed for RNA extraction. The ages of the mice were 3 to 4 months. The age of *Spalax* cannot be accurately determined, as the species cannot be bred in captivity and are instead captured in the field; hence only their weight upon capture and the years they survive in captivity can serve as a reference of their age. Accordingly, we estimate the age of the treated animals to be between 2 years old to over 10 years old. Animals were checked for tumor growth once a week until such growth was palpable. The animals were then checked two to three times a week.

For histological examination, samples were fixed in 4% paraformaldehyde dissolved in PBS; they were then dehydrated in increasing concentrations of ethanol and embedded in paraffin. Form these blocks, five-micrometer sections were cut and stained with hematoxylin and eosin for microscopic histopathological examination to assess their tumorigenesis status.

### RNA extraction and sequencing

RNA was extracted using the Qiagen miRNeasy Minikit according to the manufacturer’s protocols. In short, frozen tissues were homogenized in QIAzol lysis reagent (Qiagen). After the addition of chloroform, the solution was vortexed and centrifuged. Ethanol was added to the aqueous phase and passed through a Qiagen cleanup column. Total RNA was eluted in 30 ul volumes. Quality was assessed on an Agilent bioanalyzer, and concentrations were determined by Qubit. Sequencing was performed by the Functional Genomics Unit of the W. M. Keck Center at the University of Illinois, USA. 1μg of total RNA was used to construct the libraries using the TruSeq Small RNA Sample Prep kit (Illumina). The strand-specific RNA-Seq libraries were prepared with Illumina’s “TruSeq Stranded RNA Sample Prep” kit. The libraries were pooled in equimolar concentrations, and the pool was quantitated by qPCR and sequenced on one lane (small RNAs) or two lanes (RNA-Seq) for 101 cycles on a HiSeq2500 using a TruSeq SBS sequencing kit, version 4. Fastq files were generated and demultiplexed with bcl2fastq v1.8.4 conversion software (Illumina).

### RNA-Seq filtering and mapping

Sequencing was performed by the Functional Genomics Unit of the W. M. Keck Center at the University of Illinois. The single-end RNA-Seq data were evaluated for their quality with Fastqc (www.bioinformatics.babraham.ac.uk). The reads were trimmed from adapters and regions of low quality using Trimmomatic 0.33 [42]. The filtered fastq files were stored in the Gene Expression Omnibus repository (GSE117501). To map the *Spalax* genome, we used the genome published by Fang et al. as a reference [8]. For mice, we used the reference genome published by NCBI. The reads were mapped to their respective genomes using TopHat2 [43] along standard parameters. Sorting and indexing were done using samtools [44], and the genes were counted using htseq-count [45]. The alignments were visualized using IGV [46, 47].

### Differential expression analysis

Gene expression analysis was conducted using EdgeR [48] and DESeq2 [49]. Genes were considered differentially expressed if they had an adjusted *p* value ≤0.05. While both algorithms produced similar results, we used the consensus of both results as being differentially expressed. Functional annotation was done in R [50] using the packages topGO [51], KEGGREST [52], and biomarRt [53] for the GO [54] and KEGG [55] annotation. Gene descriptions and gene synonyms were acquired through Ensembl [56]. Gene relations were visualized using Cytoscape [46]. Additional gene information was gathered with GeneMANIA [11] and the web-based tool cBioPortal [57].

### microRNA sequencing, filtering and mapping

Libraries were constructed using the TruSeq Small RNA Sample Prep kit (Illumina, CA, USA). The libraries were quantitated by qPCR and sequenced on one lane for 101 cycles from one end of the fragments on a HiSeq 4000 using a HiSeq 4000 sequencing kit, version 1. Fastq files were generated and demultiplexed with bcl2fastq v2.17.1.14 conversion software (Illumina). Adaptors were trimmed from the reads, which were then mapped using mirDeep [58]. Differential expression analysis was conducted using DESeq2 [49], while microRNA targets and regulators were taken from OncomiRDB [59].

## Additional file

Additional file 1: **Table S1.** RNA-Expression of *Spalax*. RNA expression divided between the different samples. **Table S2**. 68 differentially expressed genes in carcinogen resistant *Spalax*. **Table S3**. miRNA analysis in *Spalax* candidate genes. Differential expression analysis of miRNAs in *Spalax* samples. **Table S4**. miRNA analysis in *Spalax* ECM genes. Differential expression analysis of miRNAs in *Spalax* samples of ECM related genes. **Table S5**. Expression values of Fanconi Anemia genes. Differential expression analysis for Fanconi Anemia genes in *Spalax*. **Table S6**. Differential expression analysis of *Spalax* UIS vs tumor. A |log2FC| ≥ 2 is a threshold for highly differentially expressed genes. We filtered the genes that belong to these categories: cell cycle, citrate cycle, immune system, extracellular matrix (ECM), hypoxia response, and degenerative diseases. **Table S7**. Differentially expressed genes in fibrosarcomas and granulomas. **Table S8**. Differentially expressed genes in granulomas only. **Table S9**. Differentially expressed genes in fibrosarcomas only. (XLSX 231 kb)

## Abbreviations

3MCS: 3-methylcholanthrene, a chemical carcinogen
DEG: Differetially expressed gene
EMC: Extracellular matrix
IS: Injected, and treated side of the animals neck
NME2: Nucleoside diphosphate alkylase 2
ROS: Reactive oxygen species
SSTR4: Somatostatin Receptor 4
TGFβ1: Transforming growth factor β1
UCP1: Mitochondrial uncoupling protein
UIS: Uninjected, and untreated side of the animals neck
